# Technologienutzung im Alter: Zusammenhänge zwischen Akzeptanz, Kompetenz, Kontrolle, Interesse und sozialen Indikatoren bei Personen über 60 Jahre

**DOI:** 10.1007/s00391-023-02225-9

**Published:** 2023-09-05

**Authors:** Drin Ferizaj, Luis Perotti, Rebecca Dahms, Anika Heimann-Steinert

**Affiliations:** Department of Geriatrics and Medical Gerontology, Charité – Universitätsmedizin Berlin, corporate member of Freie Universität Berlin and Humboldt-Universität zu Berlin, Berlin, Deutschland

**Keywords:** Technikbereitschaft, Digitale Kluft, Ältere Menschen, Informations- und Kommunikationstechnologien, Strukturgleichungsmodell, Technology readiness, Digital divide, Older adults, Information and communication technologies, Structural equation model

## Abstract

**Hintergrund:**

Neben den soziodemografischen Faktoren spielen handlungstheoretische Konstrukte wie die Technikakzeptanz oder Technikkompetenz eine wichtige Rolle bei der Techniknutzung.

**Ziel der Arbeit:**

Innerhalb der vorliegenden Studie wurde untersucht, wie die Techniknutzung mit soziodemografischen Faktoren und handlungstheoretischen Konstrukten und Technikinteresse zusammenhängt.

**Materialien und Methoden:**

Im Zeitraum von 2014 bis 2020 wurden Daten von 585 Studienteilnehmenden im Alter über 60 Jahre aus 14 Erhebungen gesammelt. Zur Erklärung der Zusammenhänge wurde ein Strukturgleichungsmodell durchgeführt.

**Ergebnisse:**

Das Strukturgleichungsmodell mit den Kovariaten Erhebungsjahr, Alter, Geschlecht und Bildung (*n* = 585) ergab den folgenden Fit: Comparative Fit Index (CFI) =0,918, Tucker-Lewis-Index (TLI ) = 0,894, Root Mean Square Error of Approximation (RMSEA) = 0,056 [95 %-Konfidenzintervall: 0,050-0,063], Standardized Root Mean Square (SRMR) = 0,079, χ^2^ = 3051,936 (*p* < 0,001), χ^2^-Test/Freiheitsgrade (df) = 18,499. Die stärksten Zusammenhänge mit der Techniknutzung zeigten sich bei der Technikakzeptanz und der Technikkompetenz. Zudem zeigte die Technikkompetenz eine signifikante Verknüpfung mit dem Technikinteresse. Das Geschlecht und das Technikinteresse standen nicht in Verbindung zur Techniknutzung, stattdessen wurde beobachtet, dass Männer höhere Ausprägungen in Technikakzeptanz, Technikkontrolle, Technikkompetenz und Technikinteresse hatten.

**Diskussion:**

Die Berücksichtigung der Technikkompetenzüberzeugungen spielt eine wesentliche Rolle für ein umfassendes Verständnis der Techniknutzung und des Technikinteresses älterer Personen. Darüber hinaus wurden geschlechtsspezifische Unterschiede in den handlungstheoretischen Konstrukten und dem Technikinteresse im Kontext der digitalen Kluft aufgezeigt.

## Abstract (en)

**Background:**

In addition to sociodemographic factors, action-theoretical constructs, such as technology acceptance and competence play an important role in technology use.

**Objective:**

This study aimed to examine the associations between technology use, sociodemographic factors, action-theoretical constructs, and technology interest.

**Material and methods:**

Data were collected from 585 study participants aged over 60 years from 14 surveys conducted between 2014 and 2020. A structural equation model was used to explain the relationships.

**Results:**

The structural equation model with covariates of survey year, age, gender, and education (*n* = 585) yielded the following fit indices: comparative fit index (CFI) = 0.918, Tucker-Lewis index (TLI) = 0.894, Root Mean Square Error of Approximation (RMSEA) = 0.056 [95 % confidence interval: 0.050–0.063], Standardized Root Mean Square Residual (SRMR) = 0.079, χ2 = 3051.936 (*p* < 0.001), χ2/degrees of freedom (df) = 18.499. The strongest associations with technology use were found for technology acceptance and competence. Additionally, technology competence showed a significant association with technology interest. Gender and technology interest were not related to technology use but it was observed that men had higher levels of technology acceptance, control, competence, and interest.

**Discussion:**

Taking technology competence beliefs into account plays a crucial role in understanding the technology usage and interest of older individuals. Additionally, gender-specific differences in the theoretical constructs of action and interest in technology have been revealed in the context of the digital divide.

## Hintergrund

Im Zuge der digitalen Revolution haben Technologien nahezu alle privaten und professionellen Lebensbereiche verändert und bieten Möglichkeiten zur Unterstützung und zur Erweiterung von Kompetenzen. Nichtsdestotrotz werden die Potenziale von Technologien nicht in allen Bevölkerungsgruppen gleichermaßen wahrgenommen [17, 29]. Insbesondere ältere Personen, Frauen und Menschen mit niedrigem formalen Bildungsabschluss werden häufiger im digitalen Abseits verortet [14, 16, 17, 21]. Neben den soziodemografischen Faktoren werden zur Erklärung der Nutzung von Technologien handlungstheoretische Konstrukte wie die Technikakzeptanz, Kontroll- und Kompetenzüberzeugungen herangezogen [10]. Die Technikakzeptanz, worunter man die subjektive Bewertung von Technologien versteht [31], konnte bereits in zahlreichen Untersuchungen als zentraler Faktor zur Vorhersage von Techniknutzung nachgewiesen werden [4, 13, 18]. Bei der Nutzung von digitalen Technologien spielen zusätzlich Kontrollüberzeugungen eine wichtige Rolle. Diese beziehen sich auf internale Attributionsmuster, die die Möglichkeit von Ergebnissen in technologiebezogenen Situationen beschreiben [24]. Technikkompetenzüberzeugungen beziehen sich auf die subjektiv eingeschätzten Handlungsmöglichkeiten im Umgang mit Technologien [24]. Die beschriebenen handlungstheoretischen Konstrukte sind miteinander korreliert und hängen ebenfalls mit demografischen Variablen zusammen. So wurde gezeigt, dass ein höheres Alter negativ mit der Technikakzeptanz und Technikkompetenz korreliert ist [24]. Darüber hinaus wurden Geschlechtsunterschiede festgestellt, wobei Männer höhere Werte in Bezug auf Technikakzeptanz, Technikkompetenz und Technikkontrolle aufweisen [16, 22, 24]. Der formale Bildungsabschluss war schwach positiv mit technologiebezogenen Kompetenz- und Kontrollüberzeugungen korreliert [24]. Neben den handlungstheoretischen Konstrukten spielt auch das Technikinteresse eine bedeutende Rolle bei der Nutzung von Technologien durch ältere Personen [7, 12]. Empirische Befunde zeigen, dass das Technikinteresse negativ mit dem Alter und der Technikangst älterer Menschen zusammenhängt [11]. Darüber hinaus konnte nachgewiesen werden, dass das verringerte Technikinteresse sowie die begrenzte Techniknutzung mit der Tendenz älterer Personen einhergehen, ihre technologischen Fähigkeiten zu unterschätzen [23].

Durch die starke Verbreitung und Diffusion von Technologien in der Gesellschaft wurden in den letzten Jahrzehnten Veränderungen in den Nutzungsmustern aller Altersgruppen beobachtet. Dabei können über alle Altersgruppen hinweg steigende Nutzungszahlen verzeichnet werden [17]. Allerdings berücksichtigen querschnittliche Untersuchungen zumeist lediglich Nutzungshäufigkeiten und weniger den Zusammenhang zwischen handlungstheoretischen Konstrukten, Technikinteresse und Technologienutzung [2, 21, 25]. Dabei werden bei digitalen Technologien umfassende Kompetenzen der Nutzenden benötigt, damit diese effektiv eingesetzt werden können. Dabei spielt das Innovativeness-Needs-Paradoxon eine besondere Rolle, bei dem bestimmte Personengruppen, die potenziell von digitalen Innovationen profitieren könnten, nicht über die erforderlichen Fähigkeiten verfügen, um sie zu nutzen [17].

Vor diesem Hintergrund zielt die folgende Untersuchung darauf ab, ein umfassenderes Verständnis für die Zusammenhänge zwischen Technikinteresse, handlungstheoretischen Konstrukten, demografischen Variablen und der Nutzung von Informations- und Kommunikationstechnologien (IKT) bei älteren Menschen zu gewinnen. Insbesondere soll untersucht werden, inwieweit Technikinteresse und handlungstheoretische Konstrukte die Nutzung von Technologien unter Berücksichtigung soziodemografischer Faktoren beeinflussen.

## Forschungsfragen

In der vorliegenden Erhebung soll daher untersucht werden, ob die Nutzung digitaler Technologien durch die beschriebenen Variablen und Konstrukte erklärt werden kann. Die folgenden Zusammenhänge werden angenommen:

1. Technikakzeptanz, Technikkontrolle und Technikkompetenz hängen positiv mit der Nutzung digitaler Technologien zusammen. Zudem werden höhere Nutzungsraten abhängig vom Erhebungsjahr erwartet.
2. Es besteht eine positive Korrelation zwischen Technikakzeptanz, Technikkontrolle und Technikkompetenz. Zudem wird erwartet, dass männliches Geschlecht in allen drei Domänen mit höheren Ausprägungen assoziiert ist.
3. Technikinteresse hängt positiv mit der Techniknutzung und negativ mit dem Alter zusammen. Zudem wird angenommen, dass Technikkompetenz, aufgrund der konzeptuellen Nähe zur Technikängstlichkeit [8, 11], das Technikinteresse vorhersagt. Außerdem wird gemäß der digitalen Kluft ein höheres Technikinteresse für Männer und jüngere Personen vermutet [17].
4. Der formale Bildungsstand hängt positiv mit der Nutzung digitaler Technologien, der Technikkompetenz und der Technikkontrolle zusammen.

## Methoden

### Stichprobenbeschreibung

Im Rahmen dieser Untersuchung wurden die Erhebungsdaten von Proband*innen aus 14 Erhebungen in einen Datensatz zusammengeführt. Die Daten wurden von einer Forschungsgruppe der Charité – Universitätsmedizin Berlin im Zeitraum zwischen 2014 und 2020 erhoben. Die Daten wurden zur Baseline der jeweiligen Untersuchung erhoben, wobei es sich bei den Studien um nichtklinische Machbarkeits- und Wirksamkeitsuntersuchungen von technologischen als auch robotischen Systemen handelt. Die Einschlusskriterien für alle Studien bezogen sich auf das Alter (jeweils über 60 Jahre) und auf den kognitiven Zustand der Proband*innen (keine schweren kognitiven Störungen und keine gesetzliche Betreuung). Die Anzahl der Versuchsproband*innen der einzelnen Studien variiert zwischen 7 und 139. Die Gesamtstichprobe umfasst 585 Versuchspersonen (53 % männlich) im Alter von 60 bis 93 Jahren (*M* = 70,9 ± 6,4) aus dem Großraum Berlin-Brandenburg (Tab. 1).

**Table 1:** 

Soziodemografische Faktoren	Deskriptive Statistik
Stichprobengröße, *n*	585
*Geschlecht, n (%)*	
Weiblich	275 (47 %)
Männlich	310 (53 %)
Alter, *M*	70,9 ± 6,4
*Bildungsstand, n (%)*	
Kein Abschluss	3 (0,07 %)
Hauptschulabschluss	49 (11,4 %)
Realschulabschluss	97 (22,56 %)
Abitur	47 (10,93 %)
Fachhochschulabschluss	74 (17,21 %)
Universitätsabschluss	160 (37,21 %)

Die Angaben zum Bildungsstand lagen bei 430 Personen vor

### Messinstrumente

#### Selbstentwickelter Fragebogen zur Erfassung von Nutzung digitaler Technologien und Technikinteresse

Die Häufigkeit der Nutzung von technologischen Basisgeräten wurde mit einem selbsterstellten Fragebogen erfasst. Die Proband*innen wurden gebeten, auf einer 4‑stufigen Likert-Skala (1: „nie“ bis 4: „häufig“) anzugeben, wie häufig sie im Alltag einen Computer, das Internet oder ein Smartphone nutzen. Der Fragebogen zur Erfassung von Nutzung von digitalen Technologien war reliabel (α = 0,55). Das Technikinteresse wurde mit einem 3‑stufigen Item erfasst („Im Allgemeinen bin ich an Technologien interessiert“; 0: „stimme eher nicht zu“ bis 2: „stimme eher zu“).

#### Kurzskala zur Erfassung der Technikbereitschaft

Zur Erfassung der Technikbereitschaft wurde die validierte *Kurzskala zur Messung der Technikbereitschaft* von Neyer [24] verwendet. Die Skala basiert auf einem Technikbereitschaftsmodell, welches drei handlungstheoretische Konstrukte umfasst, die den individuellen erfolgreichen Umgang mit Technik insbesondere im höheren Lebensalter beeinflussen. Hierbei handelt es sich um die Facetten Technikakzeptanz, Technikkompetenz- und Technikkontrollüberzeugungen. Das Messinstrument umfasst 12 Items auf einer 5‑stufigen Likert-Skala (1: stimmt gar nicht, 5: stimmt völlig), welche zur Erfassung der Subskalen sowie zur Erfassung eines Gesamt-Scores dient. Höhere Punktzahlen in der Technikbereitschaft spiegeln eine höhere subjektiv erlebte Bereitschaft zum Umgang mit Technik wider (1: „geringe Technikbereitschaft“ bis 5: „hohe Technikbereitschaft“). Der Fragebogen besitzt insgesamt gute psychometrische Eigenschaften im Hinblick auf Reliabilität und Validität (Kriteriums- und Konstruktvalidität) [24]. In dieser Erhebung wurden Cronbachs-α-Werte für die Technikkompetenz, Technikakzeptanz und Technikkontrolle von 0,92, 0,81 resp. 0,74 gefunden.

### Datenaufbereitung

Alle Fragebogen wurden innerhalb der Studien papierbasiert erhoben, welche anschließend mithilfe von Microsoft Excel (Microsoft Corporation, Redmond, WA, USA) digitalisiert wurden. Alle statistischen Analysen erfolgten mithilfe von R‑Studio (R-Version 4.0.3; Posit Software formerly RStudio, Boston, MA, USA [30]) und der Erweiterung lavaan (Version 0.6–7; Ghent University, Gent, Belgien [26]).

### Statistische Verfahren

Zur statistischen Prüfung wurde ein Signifikanzniveau von 0,05 gewählt. Es sollen Zusammenhänge zwischen der Nutzung digitaler Technologien, Kontrollüberzeugungen, Selbstwirksamkeit und Technikakzeptanz mittels Strukturgleichungsmodell (SGM) erfasst werden. Dabei werden die manifesten Kovariaten Alter, Geschlecht, Bildung und Erhebungsjahr inkludiert. Die folgenden Fit-Indizes werden zur Bewertung des Modell-Fit herangezogen: *χ*^*2*^-Teststatistik, *χ*^*2*^-Test/Freiheitsgrade, Comparative Fit Index (CFI), Tucker Lewis Index (TLI), Root Mean Square Error of Approximation (RMSEA), inklusive 90 %-Konfidenzintervall, und Standardized Root Mean Square Residual (SRMR) [28]. Zur Schätzung der Parameter wurde die Maximum-Likelihood-Methode gewählt. Fehlende Daten im SGM wurden mittels des Full-Information-Maximum-Likelihood-Algorithmus imputiert. Fehlende Werte im Rahmen dieser Untersuchung sind blockweise aufgetreten, da die erhobenen Items nicht in allen Untersuchungen, die diesem Datensatz zugrunde liegen, erfasst wurden.

## Ergebnisse

### Deskriptive Beschreibung

**Figure 1:**
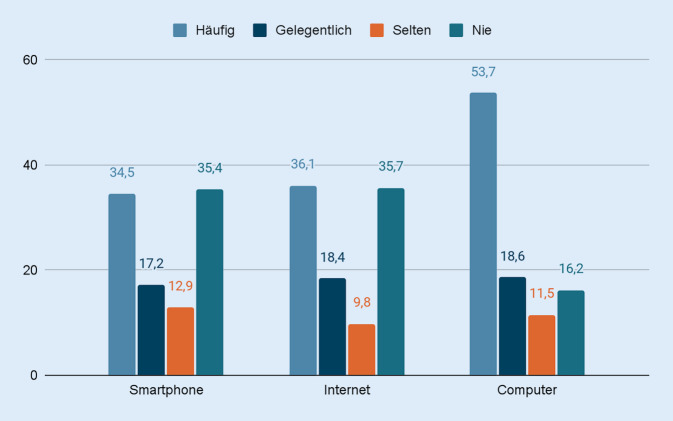

Etwa die Hälfe aller Proband*innen (53,7 %) gab an, den Computer häufig zu verwenden, während 16,2 % der Versuchspersonen angaben, keinen Computer zu verwenden. Eine häufige Nutzung des Internets wurde von 48,8 % der Proband*innen berichtet, wohingegen 10,7 % der befragten Personen das Internet nach eigenen Angaben nicht nutzten. Prozentual gesehen waren die angegebenen Nutzungshäufigkeiten von Tablets (häufige Nutzung: 36,1 %) und Smartphones (häufige Nutzung: 34,5 %) niedriger als die Nutzungshäufigkeiten des Internets und des Computers (Abb. 1). Eine Übersicht der relevanten deskriptiven Ergebnisse der handlungstheoretischen Konstrukte findet sich in Tab. 2.

**Table 2:** 

Variablen	*M*	*SD*	*n*
TB-Akz	3,4	1,0	414
TB-Komp	3,7	1,0	551
TB-Kont	3,8	0,8	444

*M* Arithmetisches Mittel, *SD* Standardabweichung, *TB* Technikbereitschaft, *TB-Akz* Technikakzeptanz, *TB-Komp* Technikkompetenz, *TB-Kont* Technikkontrolle

### Ergebnisse des SGM

**Figure 2:**
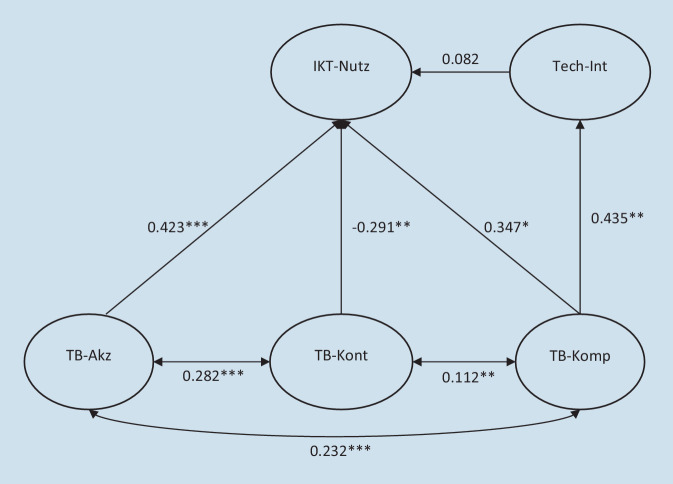

Für das SGM wurde der vollständige Datensatz verwendet (*n* = 585), und die Ergebnisse der latenten Konstrukte sind in Abb. 2 dargestellt. Das untersuchte Modell weist den folgenden Modell-Fit auf: CFI = 0,918, TLI = 0,894, RMSEA = 0,056 [95 %-Konfidenzintervall: 0,050–0,063], SRMR = 0,079, *χ*^*2*^ = 3051.936 (*p* < 0,001), *χ*^*2*^-Test/df = 18.499. Das Strukturmodell zeigt, dass höhere Werte in der Techniknutzung signifikant mit höheren Werten in der Technikkompetenz (β = 0,347, *p* = 0,022) und Technikakzeptanz (β = 0,423, *p* < 0,001), aber negativ mit der Technikkontrolle (β = −0,291, *p* = 0,007) zusammenhängen. Die IKT-Nutzung zeigte einen signifikanten negativen Zusammenhang mit dem Alter (β = −0,037, *p* < 0,001), während es keinen signifikanten Zusammenhang zwischen IKT-Nutzung und Geschlecht gab (β = 0,084, *p* = 0,527). Zudem war die IKT-Nutzung signifikant positiv mit höherem Bildungsstand (β = 0,073, *p* = 0,015) und dem Erhebungsjahr (β = 0,211, *p* < 0,001) assoziiert. Des Weiteren wurden positive Zusammenhänge zwischen Technikkompetenz und Technikkontrolle (β = 0,112, *p* < 0,001) sowie Technikakzeptanz (β = 0,232, *p* < 0,001) gefunden. Zudem war Technikakzeptanz mit Technikkontrolle signifikant positiv assoziiert (β = 0,282, *p* < 0,001). Die Technikkompetenz zeigte einen signifikant negativen Zusammenhang mit dem Alter (β = −0,033, *p* = 0,000), weiblichem Geschlecht (β = −0,427, *p* = 0,000) und einen positiven Zusammenhang mit höherem Bildungsstand (β = 0,065, *p* = 0,016). Die Technikakzeptanz zeigte eine signifikante positive Verbindung mit höherem Bildungsstand (β = 0,077, *p* = 0,016) und stand in signifikant negativer Beziehung mit weiblichem Geschlecht (β = −0,302, *p* < 0,001). Der Zusammenhang der Technikakzeptanz mit dem Alter war nicht signifikant (β = −0,011, *p* = 0,059). Die Technikkontrolle zeigte einen signifikant positiven Zusammenhang mit höherem Bildungsstand (β = 0,084, *p* = 0,008) und eine signifikante negative Verbindung mit weiblichem Geschlecht (β = −0,257, *p* = 0,002). Der Zusammenhang mit dem Alter war nicht signifikant (β = −0,002, *p* = 0,698). Technikinteresse wies keine signifikante Verknüpfung mit der IKT-Nutzung auf (β = 0,082, *p* = 0,770). Jedoch wurde beobachtet, dass Technikinteresse signifikant mit der Technikkompetenz (β = 0,435, *p* = 0,001) und männlichem Geschlecht (β = 0,372, *p* = 0,007) zusammenhing. Zudem wurden keine signifikanten Zusammenhänge zwischen Technikinteresse und Alter gefunden (β = 0,012, *p* = 0,340).

## Diskussion

Ziel der vorliegenden Erhebung war es, Beziehungen zwischen Techniknutzung, Technikinteresse und handlungstheoretischen Konstrukten wie der Technikkompetenz unter Berücksichtigung soziodemografischer Faktoren zu skizzieren. Dabei wurde gezeigt, dass die Techniknutzung v. a. mit der Technikakzeptanz und der Technikkompetenz zusammenhängt, während soziodemografische Variablen wie Alter und Bildungsstand nur eine geringe Rolle spielen. Zudem wurde gezeigt, dass das Erhebungsjahr positiv mit der Techniknutzung zusammenhängt, was im Kontext der zunehmenden Diffusion von Technologien verstanden werden kann [17]. Die Technikakzeptanz war der stärkste Prädiktor des Nutzungsverhaltens, was empirisch bereits häufig berichtet wurde [4, 13, 20]. Zudem wurde keine signifikante Beziehung zwischen der Nutzung digitaler Technologien und dem Geschlecht gefunden, jedoch wurde gezeigt, dass Männer höhere Ausprägungen in der Technikakzeptanz, Technikkompetenz und Technikkontrolle aufwiesen. Insbesondere die Technikkompetenz spielt bei Nutzungsmustern von IKT bei älteren Personen eine zentrale Rolle. Der kompetente Umgang mit Technologien wurde in zahlreichen Modellen und Studien zur Technikakzeptanz als direkte und indirekte Determinante berücksichtigt [4, 6, 8, 20]. Des Weiteren konnte kein signifikanter Zusammenhang zwischen Technikinteresse und der Techniknutzung gefunden werden. Stattdessen wurde eine deutliche Verbindung zwischen Technikinteresse und Technikkompetenz festgestellt. Die Ergebnisse von Jokisch et al. verdeutlichen dabei die Bedeutung der subjektiven Einschätzung der technologiebezogenen Fähigkeiten für die wahrgenommene Nützlichkeit von Technologien [19]. Die wahrgenommene Nützlichkeit hängt dabei zentral mit dem Technikinteresse zusammen. So zeigten Mitzner et al., dass ältere Personen Technologien positiv bewerteten und an deren Nutzung interessiert waren, sofern diese auf ihre individuellen Bedürfnisse abgestimmt und die Vorteile der Nutzung ersichtlich waren [23]. Zudem konnte in dieser Erhebung gezeigt werden, dass Technikakzeptanz und Technikkompetenz positiv miteinander zusammenhängen. Empirisch konnte bereits nachgewiesen werden, dass die Technikkompetenz stark mit den Facetten der Technikakzeptanz wie der wahrgenommenen Nützlichkeit, Einfachheit der Nutzung und Nutzungsintention zusammenhängt und im höheren Alter von zentraler Bedeutung ist [18]. Im Rahmen dieser Untersuchung konnte ein sehr schwacher negativer Zusammenhang zwischen dem Alter und der Technikkompetenz gefunden werden, was Hinweise auf die Stabilität des Konstrukts der Technikkompetenz geben kann.

Die Ergebnisse der vorliegenden Untersuchung legen nahe, dass eine hohe interne Technikkontrolle mit einer geringeren selbstberichteten IKT-Nutzung einhergeht. Personen mit umfangreicher Erfahrung im Umgang mit technologischen Geräten können möglicherweise ihre Kontrollmöglichkeiten realistischer einschätzen. Dennoch steht dieses Ergebnis im Widerspruch zu dem hierarchisch aufgebauten Modell des geplanten Verhaltens von Ajzen [1] und den darauf aufbauenden Technologieakzeptanzmodellen [4]. Da die technologiebezogene Selbstwirksamkeit konzeptuell sehr starke Überschneidungen mit den Kompetenzüberzeugungen aufweist, könnte es sein, dass die Techniknutzung im Rahmen der vorliegenden Studie größtenteils durch die Technikkompetenz und weiteren Variablen erklärt wird. Zudem ist es möglich, dass weitere Mediatorvariablen wie die Technikexpertise nicht in das Modell integriert wurden. Personen mit weniger Technikexpertise könnten demnach digitale Technologien stärker vermeiden [19] und bestehende Kontrollüberzeugungen aufgrund des Mangels an korrektiven Erfahrungen nicht anpassen. Ein zusätzlicher bedeutender Faktor, der sowohl im Rahmen der digitalen Kluft als auch in diesem Zusammenhang eine relevante Rolle spielen kann, besteht in der Bedrohung durch Stereotype. Es wurde festgestellt, dass ältere Personen ihr subjektives Alter nach der Nutzung von unbekannten Technologien höher eingeschätzt haben als nach der Verwendung von bekannten Technologien [3].

Die Techniknutzung und die handlungstheoretischen Konstrukte wiesen nur eine geringfügige Korrelation mit Alter und Bildung auf. Jüngere Personen und solche mit höherem Bildungsabschluss gaben an, dass sie technische Geräte häufiger nutzen. Diese Ergebnisse stimmen mit früheren Forschungsergebnissen überein [4, 8]. Das Geschlecht zeigte keinen signifikanten Zusammenhang mit dem IKT-Nutzungsverhalten. Jedoch zeigten Männer höhere Werte in Bezug auf die Akzeptanz von Technologie sowie die Technikkompetenz, Technikkontrolle und Interesse an Technologie. Es wurde festgestellt, dass ältere Männer das Internet häufiger nutzen als Frauen. Dies könnte darauf zurückzuführen sein, dass Frauen aufgrund geschlechtsspezifischer Sozialisation und traditioneller Rollenbilder möglicherweise weniger Vertrauen in ihre Fähigkeiten im Umgang mit Technologien haben und dementsprechend weniger biografische Erfahrungen im Bereich Technik aufweisen. Durch diesen selbstverstärkenden Prozess kann sowohl die Nutzung als auch das Interesse an Technologien gehemmt werden [10, 27].

### Limitationen

Die vorliegende Untersuchung weist einige Limitationen auf. Es wurden querschnittliche Daten aus verschiedenen Erhebungswellen analysiert, die zwar theoretisch begründete Zusammenhänge aufzeigen können, jedoch keine kausalen Schlüsse ermöglichen. Zudem ist die Generalisierbarkeit der Ergebnisse aufgrund des hohen Anteils von Personen mit einem Hochschulabschluss eingeschränkt. Die Daten wurden im Rahmen von technologischen Machbarkeitsstudien gesammelt, was darauf hinweisen könnte, dass eine selektive Stichprobe mit einer höheren Affinität zur Technologie vorliegt. Darüber hinaus wurde das Technikinteresse nur anhand eines einzelnen Items erfasst, wodurch die diversen technologischen Interessen älterer Personen nicht adäquat abgebildet werden [23].

### Konklusion

Die vorliegende Untersuchung beleuchtet die Relevanz der Technikkompetenz und Technikakzeptanz bei der Nutzung von Technologien bei älteren Personen. Es zeigt sich eine enge Verbindung zwischen dem Interesse an Technik und den Kompetenzüberzeugungen. Die Ergebnisse von Mitzner et al. verdeutlichen, dass ältere Personen dazu neigen, ihre Fähigkeiten in technologiebezogenen Situationen zu unterschätzen und weniger Selbstvertrauen aufweisen [21]. Zukünftige Forschung sollte jedoch sowohl die Techniknutzung als auch die technischen Kompetenzen differenzierter betrachten. Dabei sollten vorhandene technologische Wissensbestände älterer Menschen berücksichtigt werden. Ein umfassendes Verständnis darüber, wie ältere Personen Technologien nutzen und welche Funktionen sie verwenden, ist entscheidend, um Unterschiede in der Techniknutzung zu verstehen. Bezüglich der Technikkompetenz wäre es wünschenswert, Konzepte wie die Computer- und Internetselbstwirksamkeit heranzuziehen [5, 19]. Langzeitstudien könnten den Erwerb von Technikkompetenzen bei älteren Personen untersuchen und die Auswirkungen von Selbstwirksamkeitstrainings auf Kompetenzüberzeugungen und Bewertungen von Technologien messen. In Bezug auf das Technikinteresse wäre es ratsam, spezifisches Interesse an Technologien abzufragen, da ältere Personen Technologien insbesondere dann nutzen, wenn persönliche Bedürfnisse adressiert werden [15, 23].

Geschlecht zeigt deutliche Zusammenhänge mit Technikkompetenz, Technikakzeptanz und Technikinteresse. Zukünftige Untersuchungen sollten instrumentelle und soziale Internetaktivitäten erfassen, da geschlechtsspezifische Unterschiede in den technologischen Nutzungsmustern und -präferenzen bestehen [12, 27]. Bei der Rekrutierung von Teilnehmern sollten gezielt Gruppen einbezogen werden, die von der digitalen Kluft betroffen sind, wie z. B. ältere Frauen mit geringem Bildungsabschluss [9].

## Fazit für die Praxis

- Aus der vorliegenden Untersuchung kann die zentrale Rolle der Technikkompetenz abgeleitet werden. Zur Überbrückung der digitalen Kluft wäre es wünschenswert, den sicheren Umgang mit Technologien älterer Personen zu stärken.
- Durch die zunehmende Diffusion von Technologien im Alltag ist es wichtig, älteren Personen einen inklusiven geschlechtssensiblen Zugang zu gewähren. Dabei kann unter Berücksichtigung von technikbiografischen Erfahrungen und Sozialisationsprozessen an der Technikakzeptanz und der Technikkompetenz angesetzt werden.
- Technologiebezogene Wirksamkeitstrainings könnten als Schritt dienen, um Kompetenzen und Kontrollerleben älterer Personen zu stärken und ihr Interesse zu fördern.
